# Association between Organochlorine Pesticides and Vitamin D in Female Subjects

**DOI:** 10.3390/biomedicines11051451

**Published:** 2023-05-15

**Authors:** Edwina Brennan, Alexandra E. Butler, Manjula Nandakumar, Daniel S. Drage, Thozhukat Sathyapalan, Stephen L. Atkin

**Affiliations:** 1 School of Medicine, Royal College of Surgeons in Ireland-Medical University of Bahrain, Busaiteen 15503, Bahrain; abutler@rcsi.com (A.E.B.); mnandakumar@rcsi.com (M.N.); satkin@rcsi.com (S.L.A.); 2 School of Geography, Earth and Environmental Sciences, University of Birmingham, Edgbaston, Birmingham B15 2TT, UK; d.s.drage@bham.ac.uk; 3 Queensland Alliance for Environmental Health Sciences, The University of Queensland, 39 Kessels Road, Brisbane, QLD 4108, Australia; 4 Hull York Medical School, University of Hull, Hull HU6 7RX, UK; thozhukat.sathyapalan@hyms.ac.uk

**Keywords:** Organochlorine pesticides (OCPs), organic pollutants, vitamin D, cholecalciferol

## Abstract

In human population studies, organochlorine pesticides (OCPs) have been linked to vitamin D deficiency. Therefore, this study examined the association between OCPs, vitamin D_3_ (cholecalciferol, 25(OH)D_3_), and the active metabolite 1,25-dihydrovitamin D_3_ (1,25(OH)_2_D_3_) in a cohort of non-obese women. The serum samples of 58 female participants (age—31.9 ± 4.6 years; body mass index (BMI)—25.7 ± 3.7 kg/m^2^) were screened for 10 indicator OCPs. 25(OH)D_3_ and 1,25(OH)_2_D_3_ levels were determined via isotope dilution liquid chromatography tandem mass spectrometry. In this cohort, the 25(OH)D_3_ and 1,25(OH)_2_D_3_ levels were 22.9 ± 11.2 ng/mL and 0.05 ± 0.02 ng/mL, respectively, with 28 participants classified as 25(OH)D_3_-deficient (<50 nmol/L). In the study cohort, no correlations were found between individual or total OCPs (ƩOCPs) and 25(OH)D_3_. p,p′-dichlorodiphenyldichloroethylene (DDE) and ƩOCPs correlated positively with 1,25(OH)_2_D_3_, with the latter being negatively correlated with estimated glomerular filtration rate (eGFR). In women with sufficient 25(OH)D_3_ levels, p,p′-dichlorodiphenyltrichloroethan (DDT) was positively correlated with 1,25(OH)_2_D_3_, whilst in the deficient group, hexachlorobenzene (HCB) and p,p′-(DDE) were positively correlated with 1,25(OH)_2_D_3_, β-Hexachlorocyclohexane (HCH) was positively correlated with 25(OH)D_3_, and none of the OCPs were associated with measures of renal function. Overall, OCPs and ƩOCPs were not associated with 25(OH)D_3_, suggesting that they are unrelated to vitamin D deficiency, but p,p′-DDE and ƩOCPs correlated positively with active 1,25(OH)_2_D_3_, while ƩOCPs correlated negatively with eGFR, suggesting a possible renal effect. Analysis of vitamin D deficiency revealed an association between β-HCH and 25(OH)D_3_, and between HCB and p,p′-DDE and 1,25(OH)_2_D_3_, suggesting that OCP effects may be enhanced in cases of vitamin D deficiency.

## 1. Introduction

Organochlorine pesticides (OCPs) are a group of chlorinated hydrocarbons that were once used extensively in agriculture and mosquito control and have since been termed persistent organic pollutants (POPs) due to their high toxicity, slow degradation, and bioaccumulation [1]. OCPs are highly persistent in the environment and can accumulate in the fatty tissues of animals and humans [2,3]. They can also be transported over great distances in the air and water, leading to the contamination of soil and water sources far from the OCPs’ original application site [4]. Due to the potential health risks associated with OCPs, many countries have banned or severely restricted their use in recent years [5]. However, these chemicals can persist in the environment for decades [4] and may still be found in some food and water sources.

Exposure to OCPs has been linked to a variety of health issues. OCPs have been linked to an increased risk of breast [6] and prostate [7] cancer, and lymphoma [8]. OCPs have been linked to reproductive problems in both men and women, including decreased fertility [9,10] and miscarriages, particularly with respect to hexachlorocyclohexane (HCH) [11], and prenatal exposure to α-HCH, β-HCH, ο,p′-dichlorodiphenyldichloroethane (DDD), and p,p′-DDD has been associated with an increased risk for neural tube birth defects [12]. OCPs have been linked to neurological problems, which they are thought to contribute to via their accumulation in the body over time, potentially leading to Parkinson’s disease (HCH) [13] and Alzheimer’s disease (p,p′-dichlorodiphenyldichloroethylene (DDE)) [14]. OCPs have been linked to immune system disorders, with dichlorodiphenyltrichloroethane (DDT) causing the dysregulation of interleukin 6 (IL-6), leading to chronic inflammation [15], and having been related to oxidative stress and immune suppression [16], thus increasing susceptibility to infections and other diseases. OCPs have been classed as endocrine disruptors that can cause developmental problems in children (DDT) [17] and reproductive problems in adults [9]. In addition, low doses of OCPs have been strongly linked to various chronic diseases including diabetes [18] and cardiovascular diseases [19].

OCPs may have several mechanisms of action that contribute to their reported diverse effects. OCPs function by binding to specific target sites in the body, including binding to and blocking the GABAA receptors of sodium channels in the brain [20]. By binding to these sites, they disrupt the normal functioning of the nervous system, leading to the overstimulation or inhibition of nerve impulses. OCPs can also interfere with the normal function of the endocrine system by disrupting the production, release, transport, and metabolism of hormones at several sites of action such as the hypothalamus (increasing gonadotropin-releasing hormone mRNA levels), pituitary glands (affecting luteinizing hormone release), and ovaries (decreased sex steroid hormone production) [20].

Vitamin D deficiency is associated with various health conditions such as osteoporosis, cancer, cardiovascular disease, autoimmune diseases, and increased mortality [21,22]. In addition, vitamin D deficiency is associated with an increased incidence of type 2 diabetes [23,24,25] and microvascular and macrovascular diabetes-related complications [26]. Vitamin D_3_ (cholecalciferol, 25(OH)D_3_) is endogenously produced via the UV-B irradiation of 7-dehydrocholesterol and is hydroxylated to 25(OH)D_3_ by multiple 25-hydroxylases in the liver [27]. 25(OH)D_3_ is transported to the kidneys and converted to active 1,25-dihydrovitamin D_3_ (1,25(OH)_2_D_3_) by 1 alpha hydroxylase present in the renal tubular cells [28]. It has recently been reported that extrarenal tissues may also convert 25(OH)D_3_ to 1,25(OH)_2_D_3_, although activation in renal and non-kidney tissues is regulated differently via the macrophage production of 1,25(OH)_2_D_3_ through the type 2 interferon (IFN) response [29].

There is some evidence suggesting that exposure to OCPs may be associated with lower levels of vitamin D in the body [19,30]. Population studies have suggested that OCPs, including p,p′-DDT, p,p′-DDE, and β-HCH, can cause vitamin D deficiency [31]. In one such study, Inuit people who were heavily exposed to these pesticides through their traditional diet of fish and marine mammals, causing accumulation of these pesticides, showed higher levels of OCPs in their blood and had lower levels of vitamin D [32]; however, chronobiological factors within this population are likely to play a role.

OCPs may contribute to vitamin D deficiency through their effects on vitamin D metabolism in the liver, the disruption of the conversion of 25(OH)D_3_ to 1,25(OH)_2_D_3_, interference with the function of vitamin D receptors in the body, or the impairment of calcium homeostasis [33]. It is important to note that the evidence linking OCPs and vitamin D levels is still limited, and more research is needed to fully understand the relationship between these two factors. However, the evidence still highlights the potential health risks associated with exposure to these persistent environmental pollutants. Therefore, this study was undertaken to examine the association between OCPs (pentachlorobenzene (PeCB), hexachlorobenzene (HCB), HCH, lindane, trans-chlordane, cis-chlordane, DDE, DDD, DDT, and dodecachlorooctahydro-1,3,4-metheno-1H-cyclobuta[cd]pentalene (Mirex)) and 25(OH)D_3_ and its active metabolite 1,25(OH)_2_D_3_ in a group of non-obese healthy women prior to undergoing in vitro fertilization (IVF). In addition, we sought to determine if such an association may be precipitated by the impairment of calcium homeostasis, specifically with respect to its association with calcium/calmodulin-dependent (CaM) kinases (CaMK), CaMK kinase alpha (CaMKKα), and CaM phosphodiesterase (PDE) proteins.

## 2. Materials and Methods

### 2.1. Patient Recruitment

Subjects were recruited from the Hull IVF Unit, UK, following ethical approval from The Yorkshire and The Humber NRES ethical committee, UK (approval number 02/03/043). Venesection was performed on 58 fasting, non-obese Caucasian women; samples were taken at 09:00 on day 21 of the menstrual cycle prior to IVF downregulation. Venous blood samples were centrifuged at 3500× *g* for 15 min at 4 °C, and serum was stored at −80 °C within 1 h of collection for further analysis. No participant was on any prescribed or over-the-counter medication. All participants provided written informed consent.

### 2.2. OCP Measurement

Samples were screened for 10 indicator OCPs (PeCB, HCB, HCH, lindane, trans-chlordane, cis-chlordane, DDE, DDD, DDT, and Mirex). A sum-of-OCPs (ƩOCPs) variable was calculated by adding the molar concentrations of each OCP analyzed. After vortexing the samples for 1 min, 6 mL of acetonitrile, 3 mL of milliQ water, 5 g of anhydrous MgSO_4_, and 1 g of NaCl were added along with a ceramic homogenizer. Samples were manually shaken for 1 min before being centrifuged at 4700 RPM for 8 min at 10 °C. The supernatant layer (approximately 6 mL of acetonitrile) was collected and transferred to 12 mL glass vials. An aliquot of 200 µL was collected and cleaned for non-targeted analysis (not presented here). The remainder of the extract was transferred to a 15 mL glass tube, evaporated under a gentle stream of nitrogen to near-dryness, and reconstituted in approximately 1 mL of hexane. Approximately 1 mL of >98% concentrated sulfuric acid was added, and the sample was vortexed for at least 30 s. The aqueous and hexane layers were left to separate overnight at <4 °C. The supernatant (hexane) layer was transferred directly onto a silica solid-phase extraction (SPE) cartridge (Supelco LC-Si, 3 mL/500 mg) (preconditioned with 6 mL dichloromethane (DCM) followed by 6 mL of hexane). Target compounds were eluted into a 15 mL glass collection tube using 6 mL of hexane followed by 8 mL of DCM. Samples were evaporated to near-dryness and reconstituted in 50 µL of methanol containing 2.5 ng ^13^C_12_-PCB-141 as a recovery standard. Samples were transferred to autosampler vial inserts prior to analysis.

### 2.3. Instrumental Analysis

OCPs were identified in serum samples via high-resolution gas chromatography coupled with high-resolution mass spectrometry (HRGC/HRMS). A Thermofisher TRACE 1300 gas chromatograph was coupled to a Thermofisher DFS mass spectrometer. The injector was operated in splitless mode with separation achieved on an Agilent DB-5ms column (30 m length × 0.25 mm in diameter × 0.25 µm film thickness). Experiments were conducted in multiple ion detection (MID) mode at a resolution level of 10,000 (10% valley definition). The inlet, transfer line, and source were maintained at 250 °C, 280 °C, and 280 °C, respectively. The flow rate was maintained at 1.0 mL/min. In the GC oven, an initial temperature of 80 °C was held for two minutes before being ramped up to 180 °C at 20 °C/min and held for 0.5 min. The temperature was then increased to 300 °C at 10 °C/min and held for 5 min.

### 2.4. Quality Assurance/Quality Control

The limit of reporting (LOR) was determined according to the quality of the peak shape and using a minimum signal-to-noise ratio of 10:1. Contamination was assessed via inclusion of procedural blanks (milli-Q water, n = 6) extracted in parallel with unknown samples. If a target compound was detected in a blank at below 5% of a sample, then no correction occurred. When blank concentrations were 5–25% of a respective sample, the sample was blank corrected. Linearity was established with a seven-point calibration across the range of 0.1 to 25 ng/mL for all target analytes. R2 values > 0.99 were achieved for all compounds. Analytes were identified based on retention time (<2% RSD from standard) and confirmed with reference to a second ion (HRGC/HRMS). Quantification was performed using isotope dilution mass spectrometry. All OCPs were quantified against 13C12-HCB. All data were validated by spiking 5 aliquots of bovine serum with 300 pg of target analytes. The concentrations of the spiked samples were measured, and the recoveries (measured concentration/spiked concentration × 100) were calculated. Average recoveries were 80–120% with a relative standard deviation of <15%, thus conforming to the OECD guidelines of good laboratory practice (Supplementary Table S1).

### 2.5. Vitamin D_3_ and Biochemical Parameters

Biochemical and hormonal parameters were measured as per a previously detailed method [34]. Briefly, serum 25(OH)D_3_ and 1,25(OH)_2_D_3_ levels were quantified using isotope dilution liquid chromatography tandem mass spectrometry (LC-MS/MS). Vitamin D metabolites and labelled internal standards were extracted from 250 µL of serum using supportive liquid–liquid extraction and Diels–Alder derivatization prior to LC-MS/MS analysis. Chromatographic separations were achieved using Hypersil Gold C18 column (150 × 2.1 mm; 1.9 µm) at a flow rate of 0.2 mL/min and operated in Electrospray Ionization (ESI) positive mode and analyzed using the multiple-reaction-monitoring (MRM) method. The limit of quantification (LOQ) for 1,25(OH)_2_D_3_ was 10 pg/mL and 25(OH)D_3_ 1.2 nmol/L (0.5 ng/mL) [35]. Vitamin D deficiency was defined as a cutoff of 20 ng/mL (50 nmol/L) as per the clinical practice guidelines of the Endocrine Society Task Force on Vitamin D [36].

### 2.6. CaM Proteomic Measurement

Circulating levels of CaM-associated proteins (PDE1A, CaMK2A, CaMK2B, CaMK1D, CaMK2D, CaMK1, and CaMKKα) were determined via Slow Off-rate Modified Aptamer (SOMA)-scan plasma protein measurement (Somalogic, Boulder, CO, USA), the details of which have been previously reported [37]. Briefly, EDTA plasma samples were diluted, and the following assay steps were performed: (a) Binding: analytes and primer bead–SOMAmers (fully synthetic fluorophore-labelled SOMAmer coupled to a biotin moiety through a photocleavable linker) are equilibrated. (b) Catch I: all analyte–SOMAmer complexes are immobilized on a streptavidin-substituted support. Washing steps: proteins not stably bound to primer bead–SOMAmers are removed, and bound protein is biotinylated. (c) Cleave: long-wave ultraviolet light is applied to release analyte SOMAmer complexes into the solution. (d) Catch II: analyte–SOMAmer complexes are selectively immobilized on streptavidin support via the introduced analyte-borne biotinylation. Further washing is performed to select against non-specific analyte–SOMAmer complexes. (e) Elution: denaturation is induced to disrupt analyte–SOMAmer complexes. Released SOMAmers serve as surrogates for quantification of analyte concentrations. (f) Quantification: SOMAmer complementary oligonucleotides are hybridized to custom arrays. Normalization of raw intensities, hybridization, median signal, and calibration signal were performed based on the standard samples included on each plate, as previously described [38].

### 2.7. Statistics

No previous studies were available to enable the performance of a power analysis; therefore, this pilot study was designed according to the research conducted by Birkett and Day [39]. Descriptive data are presented as mean ± standard deviation (SD) for continuous data. Potential correlations between OCPs, 25(OH)D_3_), and 1,25(OH)_2_D_3_ were examined using exploratory Spearman’s rank order correlations. A *p*-value of <0.05 was considered to indicate statistical significance. Statistical analysis was carried out using Jamovi (version 2.0.0).

## 3. Results

### 3.1. Whole-Cohort Analysis

Demographic and biochemical data for this cohort are shown in Table 1. The participants had a mean age of 31.9 ± 4.6 years and a mean body mass index (BMI) of 25.7 ± 3.7 kg/m^2^. Thyroid function and C-reactive protein (CRP) levels, constituting measures of underlying inflammation, were normal. The mean levels of 25(OH)D_3_ and 1,25(OH)_2_D_3_ were 22.9 ± 11.2 ng/mL and 0.05 ± 0.02 ng/mL, respectively. Of the 58 women recruited, 28 had a 25(OH)D_3_ level less than 20 ng/mL (50 nmol/L). The obtained levels of OCPs, 25(OH)D_3_, and 1,25(OH)_2_D_3_ are shown in Table 1.

### 3.2. OCP Levels

The detection frequency of individual OCPs ranged from 100–12%. p,p′-DDE was detected in all serum samples, making it the most abundant OCP, followed by HCB (95%), p,p′-DDT (74%), β-HCH (67%), cis-chlordane (48%), Mirex (41%), PeCB (38%), trans-chlordane (24%), o,p′-DDE (22%), and lindane (12%). The OCPs α-HCH, o,p′-DDD, and p,p′-DDD+o,p′-DDT were not detected.

### 3.3. Whole-Group Correlations

**Figure 1:**
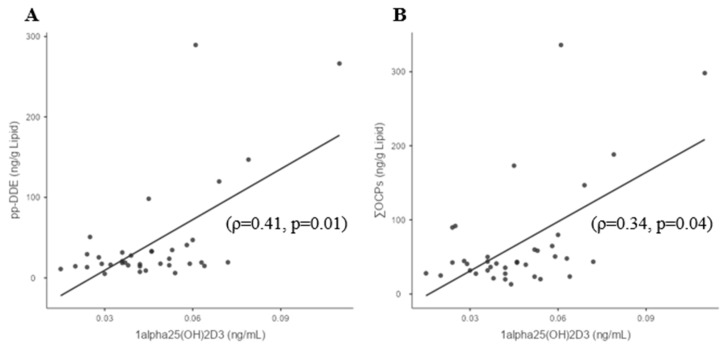

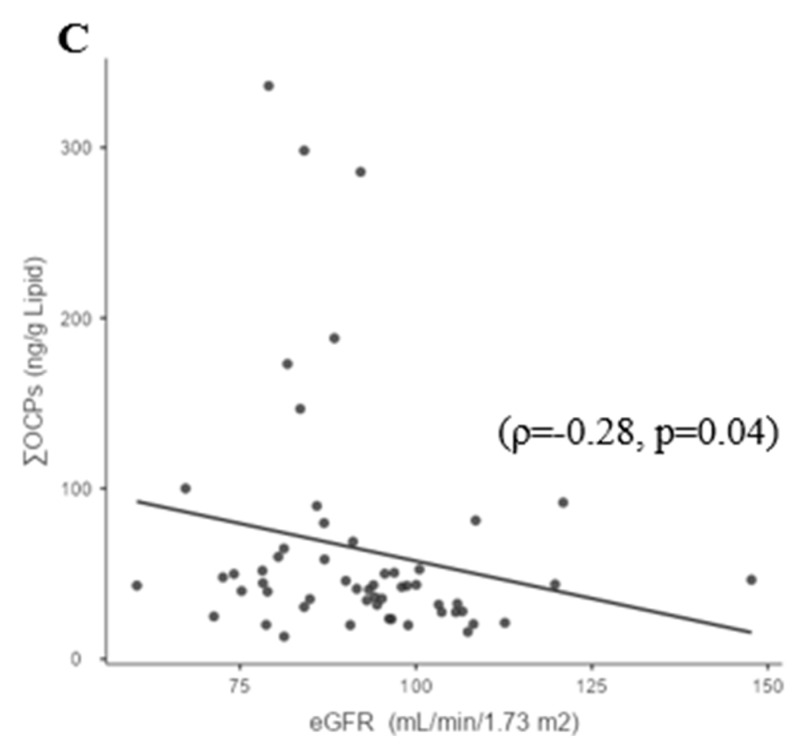
Significant correlations of OCPs with 1,25(OH)_2_D_3_ and measures of renal function in whole study cohort. (**A**) 1,25(OH)_2_D_3_ and p,p′-DDE; (**B**) 1,25(OH)_2_D_3_ and ƩOCPs; (**C**) eGFR and ƩOCPs.

In the study cohort, no correlations were found between ƩOCPs or individual OCPs and 25(OH)D_3_. The OCP p,p′-DDE and the ƩOCPs positively correlated with 1,25(OH)_2_D_3_, (ρ = 0.41; *p* = 0.01) and (ρ = 0.34; *p* = 0.04), respectively (Figure 1A,B), but no other correlations were found between individual OCPs and 1,25(OH)_2_D_3_ (Table 2). The ƩOCPs variable negatively correlated with eGFR (ρ = −0.28; *p* = 0.04) (Figure 1C). There were no correlations between p,p′-DDE or ƩOCPs with urea and creatinine.

### 3.4. Sufficient and Deficient 25(OH)D_3_ Subgroup Correlations

**Figure 2:**
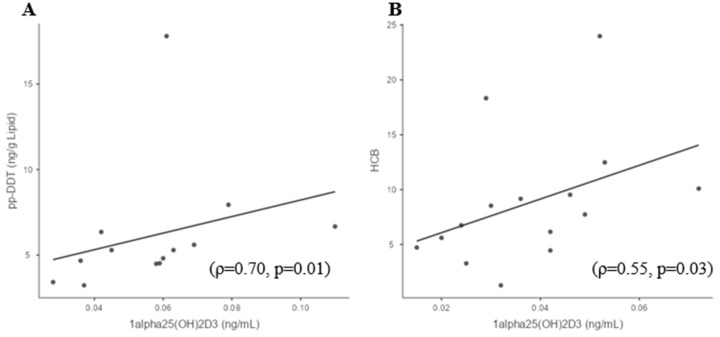

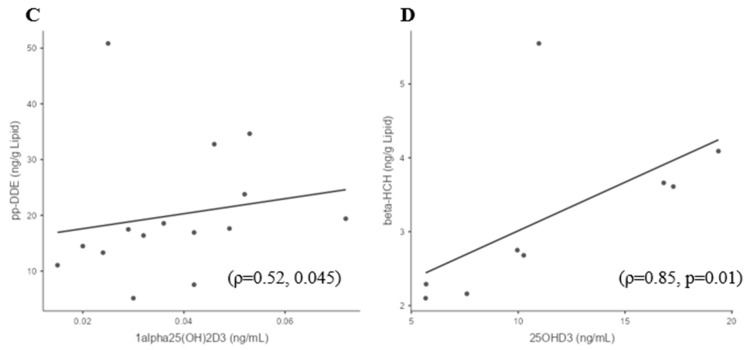
Significant correlations of OCPs with 25(OH)D_3_ and 1,25(OH)_2_D_3_ in cohort subgroups. The study cohort was divided into two subgroups based on the participants’ vitamin D levels: sufficient 25(OH)D_3_ (>20 ng/mL; >50 nmol/L) women and deficient 25(OH)D_3_ (<20 ng/mL; >50 nmol/L) women. Sufficient vitamin D subgroup—(**A**) 1,25(OH)_2_D_3_ and p,p′-DDT. Deficient vitamin D subgroup—(**B**) 1,25(OH)_2_D_3_ and HCB; (**C**) 1,25(OH)_2_D_3_ and p,p′-DDE; (**D**) 25(OH)D_3_ and β-HCH.

The study cohort was divided into two subgroups based on the participants’ vitamin D levels: sufficient 25(OH)D_3_ (>20 ng/mL; >50 nmol/L) women and deficient 25(OH)D_3_ (<20 ng/mL; <50 nmol/L) women. In the sufficient 25(OH)D_3_ women, p,p′-DDT strongly positively correlated with 1,25(OH)_2_D_3_ (ρ = 0.70; *p* = 0.01) (Figure 2A). There were no correlations found between OCPs and 25(OH)D_3_ in the sufficient 25(OH)D_3_ women subgroup. In deficient 25(OH)D_3_ women, HCB and p,p′-DDE positively correlated with 1,25(OH)_2_D_3_ ((ρ = 0.55; *p* = 0.03) and (ρ = 0.52; *p* = 0.045), respectively (Figure 2B,C)), while β-HCH strongly positively correlated with 25(OH)D_3_ (ρ = 0.85; *p* = 0.01) (Figure 2D). Calcium levels did not differ between vitamin D sufficiency (2.3 ± 0.07 mmol/L) and vitamin D deficiency groups (2.3 ± 0.06 mmol/L).

We have found that 1,25(OH)_2_D_3_ levels may be modulated through CaMK1 by polybrominated diethyl ether 153 (unpublished results); therefore, the CaM-associated proteins (PDE1A, CaMK2A, CaMK2B, CaMK1D, CaMK2D, CaMK1, and CaMKKα) (Table 1) that effect the rapid action of 1,25(OH)_2_D_3_ at the cell membrane leading to increasing intracellular calcium levels were investigated, but no associations with the individual or total OCPs, or in vitamin-D-sufficient or deficient states, were found.

## 4. Discussion

These data show that the 10 indicator OCPs and ƩOCPs analyzed do not appear to be associated with 25(OH)D_3_ but that the OCP p,p′-DDE and ƩOCPs correlated positively with active 1,25(OH)_2_D_3._ Overall, the ƩOCPs were negatively associated with a reduced eGFR level, as noted by others [40], indicating that their action may be exerted on the kidneys, as suggested by the reported association of DDT burden with chronic kidney disease [41], but this was an observational study with no mechanistic insights. An inverse relationship of OCPs, including p,p′-DDT, p,p′-DDE, and β-HCH, with vitamin D has been reported [31], but this was not seen in the data presented herein; however, this difference is potentially due to the differing study populations, as those associations were seen amongst subjects of an older age, who were Caucasian, or who suffered from chronic diseases, whereas the population analyzed in this study was young and healthy.

When the study cohort was divided into two subgroups, namely, sufficient and deficient groups based on their vitamin D levels, in the vitamin-D-sufficient 25(OH)D_3_ women, p,p′-DDT strongly positively correlated with 1,25(OH)_2_D_3_. There were no correlations found between OCPs and 25(OH)D_3_ in the vitamin-D-sufficient 25(OH)D_3_ subgroup of women. In the vitamin-D-deficient 25(OH)D_3_ women, both p,p′-DDE and HCB positively correlated with 1,25(OH)_2_D_3_. In the vitamin-D-deficient group, β-HCH strongly positively correlated with 25(OH)D_3_, suggesting that a reduction in vitamin D may signal a potential effect of both HCB and HCH, but it is unclear what those specific effects may be. The mechanism(s) behind the positive associations between OCPs and 1,25(OH)_2_D_3_ is unclear, but it has been reported that in cells exposed to p,p′-DDT and p,p′-DDE, this led to an increase in the vitamin D binding protein [42] that binds to 1,25(OH)_2_D_3_ for its activity, perhaps leading to an increase in 1,25(OH)_2_D_3_ levels.

**Figure 3:**
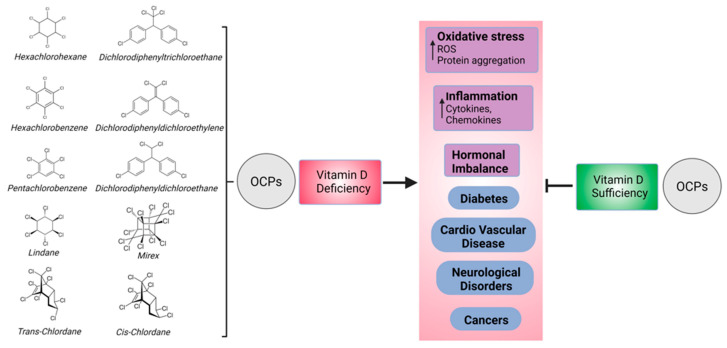
OCPs analyzed in this study. The association of OCPs with various diseases in vitamin D sufficiency and deficiency were analyzed. Vitamin D sufficiency may generally mitigate the inflammatory and oxidative stress effects and associated disorders/diseases arising due to exposure to OCPs, but in vitamin D deficiency, those effects are unmasked and aggravated.

1,25(OH)_2_D_3_ increases calcium absorption via vitamin D receptor (VDR) binding and exerts rapid action at the cell membrane, increasing intracellular calcium levels by activating protein kinase cascades that stimulate VDR-mediated transcription-enhancing steroid receptor coactivator (SRC) activity, with the overall effect of enhancing 1,25(OH)_2_D_3_-dependent interactions between VDR and SRC coactivators [43]. To investigate a possible mechanism by which OCPs may affect 1,25(OH)_2_D_3_ action, the determination of the plasma levels of CaM proteins (CaMK, CaMKK, and PDE), which may be indicative of 1,25(OH)_2_D_3_ action, was undertaken. No correlations with total or individual OCPs were found, suggesting that the OCPs impacting 1,25(OH)_2_D_3_ are not acting at the membrane level through calmodulin-associated proteins. Therefore, it is clear that 25(OH)D_3_ is converted to its biologically active form 1,25(OH)_2_D_3_ in the presence of OCPs without affecting serum calcium levels, as has been described in other animal species [44], and it is unclear how OCPs may then impair the vitamin D biological response. That the OCPs appeared to be associated with vitamin D and vitamin D sufficiency or deficiency suggests that OCPs’ effects may be mediated through vitamin D. Of particular concern, OCPs may potentiate vitamin D deficiency, which is a condition that affects over 50% of the global population [45]. Moreover, the diseases that are associated with vitamin D deficiency, such as diabetes and cardiovascular disease [21,22], are the same as those attributed to OCP exposure [19,30], suggesting that one may represent an epiphenomenon of the other. Another potential explanation is that the association between OCPs and vitamin D may be caused by indirect mechanisms. In vitro, OCPs induce inflammation and oxidative stress, with β-HCH, DDE, and Dieldrin causing the upregulation of pro-inflammatory cytokines such as tumor necrosis factor (TNF)-α, IL-1β, IL-6, nuclear factor kappa B (NF-kB), and cyclooxygenase (COX)-2 expression in OCP-treated human ovary surface epithelial cells [46]. These findings are in line with observations that DDT causes the dysregulation of IL-6 leading to chronic inflammation [15] and that DDT is related to oxidative stress and immune suppression [15,16]. This is also reflected in in vivo findings, where, in a systematic review, exposure to OCPs was positively associated with CRP, IL-1β, IL-2, and IL-10, which is indicative of dysregulated inflammatory responses; however, the underlying mechanisms remain unclear [47]. Conversely, vitamin D prevents oxidative stress, while vitamin D deficiency induces the disruption of mitochondrial function [48] and is also associated with markers of inflammation [49]. Consequently, in vitamin D sufficiency, this may generally mitigate the inflammatory and oxidative stress effects of the OCPs; however, in vitamin D deficiency, these effects have already been uncovered and indeed may be exacerbated (Figure 3), as seen with results reporting that vitamin D deficiency promotes colonic inflammation [50] and, in an animal model of acetaminophen-induced liver injury, exacerbates induced oxidative stress [51].

In addition, extrarenal tissues may also convert 25(OH)D_3_ to 1,25(OH)_2_D_3_; however, notably, activation in renal and non-kidney tissues is regulated differently, with macrophage production of 1,25(OH)_2_D_3_ through the type 2 IFN response [29]. In animal models, OCPs do not appear to affect macrophages; therefore, their influence through an extrarenal effect on 1,25(OH)_2_D_3_ appears less likely [52]. However, DDT caused an increase in IFNγ secretion [53]; increasing IFNγ may have the effect of increasing the conversion of 25(OH)D_3_ to 1,25(OH)_2_D_3_ in the extrarenal tissues.

The strengths of this study include its state-of-the-art measurement of OCPs and vitamin D (25(OH)D_3_ and 1,25(OH)_2_D_3_). Its limitations include the small number of subjects included, who were all Caucasian females, so these findings may not be generalizable to male subjects or those of different ethnicities. The low numbers compounded by the detection number may have resulted in a type-two statistical error (false negative); however, this study will potentiate the determinative power of a larger study focusing on vitamin D deficiency. OCPs have been associated with parathyroid hyperplasia leading to elevated parathyroid hormone (PTH) and calcium levels [54]; however, the calcium levels were in the normal reference range in these patients, thus obviating the need to measure PTH.

## 5. Conclusions

In conclusion, our findings show that, overall, OCPs and ƩOCPs were not associated with 25(OH)D_3_, suggesting that they are unrelated to vitamin D deficiency, and that the OCP p,p′-DDE and the ƩOCPs correlated positively with active 1,25(OH)_2_D_3_ and negatively with eGFR, suggesting a renal effect. Vitamin D deficiency unmasked an association between β-HCH and 25(OH)D_3_ and between HCB and p,p′-DDE and 1,25(OH)_2_D_3_, suggesting that OCPs’ effects may be enhanced in patients with vitamin D deficiency.

## Figures and Tables

**Table 1:** Mean and standard deviation of OCPs in the population studied.

	Female Subjects (n = 58)	
	Mean	SD
Age (years)	31.9	4.6
BMI (kg/m^2^)	25.7	3.7
CRP (mg/L)	2.6	2.5
TSH (mU/L)	2.4	2.2
Free-T3 (pmol/L)	4.8	0.7
Free-T4 (pmol/L)	11.3	1.8
25(OH)D_3_ (ng/mL)	23	11.2
1,25(OH)_2_D_3_ (ng/mL)	0.05	0.02
Calcium (mmol/L)	2.3	0.07
Urea (nmol/L)	4.66	5.21
Creatinine (nmol/L)	66	8.86
eGFR (mL/min/1.73 m^2^)	92.3	14.8
PDE1A (RFU)	789	1060
CAMK2A (RFU)	373	453
CaMK2B (RFU)	859	1423
CaMK1D (RFU)	1695	580
CaMK2D (RFU)	2379	3462
CaMK1 (RFU)	4681	1371
CaMKK α (RFU)	343	463
PeCB (ng/g Lipid)	9.12	9.1
α-HCH (ng/g Lipid)	<LOR	
β-HCH (ng/g Lipid)	4.63	4.92
HCB (ng/g Lipid)	9.81	6.5
Lindane (ng/g Lipid)	1.35	0.28
Trans-Chlordane (ng/g Lipid)	1.4	0.97
cis-Chlordane (ng/g Lipid)	1.59	0.58
p,p′-DDE (ng/g Lipid)	40.7	60.4
o,p′-DDE (ng/g Lipid)	2.23	0.32
o,p′-DDD (ng/g Lipid)	<LOR	
p,p′-DDD+o,p′-DDT (ng/g Lipid)	<LOR	
p,p′-DDT (ng/g Lipid)	4.99	2.3
Mirex (ng/g Lipid)	3.27	0.69
ƩOCPs (ng/g Lipid)	63.3	66.8

BMI: body mass index; CRP: C-reactive protein; TSH: thyroid-stimulating hormone; Free-T3: free-triiodothyronine; Free-T4: free-thyroxine; eGFR: estimated glomerular filtration rate; PDE: calcium/calmodulin-dependent phosphodiesterase; CaMK: calcium/calmodulin-dependent kinase; CaMKK α: calcium/calmodulin-dependent kinase kinase alpha; PeCB: pentachlorobenzene; α: alpha; β: beta; HCH: hexachlorocyclohexane; HCB: hexachlorobenzene; p: para; o: ortho; DDE: dichlorodiphenyldichloroethylene; DDD: dichlorodiphenyldichloroethane; DDT: dichlorodiphenyltrichloroethane; Mirex: dodecachlorooctahydro-1,3,4-metheno-1H-cyclobuta[cd]pentalene; SD: standard deviation; LOR: limit of reporting; ƩOCPs: total organochlorine pesticides.

**Table 2:** Spearman’s rho (ρ) correlations of OCPs with 25(OH)D_3_ and 1,25(OH)_2_D_3_. Data in bold indicate statistically significant correlations.

	PeCB	β-HCH	HCB	Lindane	trans-Chlordane	cis-Chlordane	o,p′-DDE	p,p′-DDE	p,p′-DDT	Mirex	ƩOCPs
25(OH)D_3_	0.289	0.174	0.101	0.393	−0.14	0.325	0.374	0.272	0.096	−0.204	0.284
	(0.192)	(0.339)	(0.502)	(0.40)	(0.648)	(0.091)	(0.258)	(0.061)	(0.595)	(0.361)	(0.051)
1,25(OH)_2_D_3_	−0.108	0.315	0.333	−0.2	−0.205	0.278	0.06	**0.408**	0.364	−0.03	**0.34**
	(0.66)	(0.14)	(0.05)	(0.92)	(0.74)	0.263	(0.89)	**(0.01)**	(0.09)	(0.92)	**(0.04)**

## Data Availability

The data presented in this study are available on reasonable request from the corresponding author.

## Supplementary Materials

The following supporting information can be downloaded at: https://www.mdpi.com/article/10.3390/biomedicines11051451/s1. Supplementary Table S1. OCP validation data.
